# TAK1-inhibitors did not reduce disease burden in a Vκ*MYC model of multiple myeloma

**DOI:** 10.1186/s13104-022-06237-3

**Published:** 2022-11-26

**Authors:** Erling Håland, Ingrid Nyhus Moen, Esten N. Vandsemb, Kristian K. Starheim

**Affiliations:** 1 CEMIR Centre of Molecular Inflammation Research, IKOM, NTNU, Trondheim, Norway; 2 Department of Clinical and Molecular Medicine, NTNU, Trondheim, Norway; 3 Department of Hematology, St. Olavs University Hospital, Trondheim, Norway

**Keywords:** Multiple myeloma, TAK1, Haematological malignancy, 5Z-7, NG25

## Abstract

**Objective:**

Multiple myeloma is a haematological malignancy characterized by proliferation of monoclonal plasma cells in the bone marrow. Development of resistance and minimal residual disease remain challenging in the treatment of multiple myeloma. Transforming growth factor-β activated kinase 1 (TAK1) has recently gained attention as a potential drug target in multiple myeloma. This study aimed at determining the in vivo effects of TAK1-inhibitors in a Vκ*MYC multiple myeloma mouse model.

**Results:**

We treated mice carrying Vκ*MYC multiple myeloma cells with the TAK1-inhibitors 5Z-7-oxozeaenol and NG25. There were tendencies towards increased survival for both inhibitors, but only NG25 prolonged survival significantly. However, this effect was limited, and no differences in disease burden were observed for any of the treatments. In conclusion, although TAK1-inhibitors might prolong survival somewhat, they do not prevent disease in the Vκ*MYC mouse model of multiple myeloma.

**Supplementary Information:**

The online version contains supplementary material available at 10.1186/s13104-022-06237-3.

## Introduction

Multiple myeloma (MM) is a haematological malignancy characterized by proliferation of abnormal monoclonal plasma cells in the bone barrow. This can lead to uncontrolled growth and cause myeloma defining events such as bone lesions, kidney failure, anaemia, and hypercalcemia [1].

There are numerous treatment options available for MM. However, most of myeloma patients endure several relapses, ultimately leading to death caused by the disease itself or by complications from treatment [2]. Common treatment strategies of MM include autologous stem cell transplantation, steroids, immunomodulatory drugs, proteasome inhibitors, monoclonal antibodies, deacetylating agents, and alkylating agents or other DNA-damaging drugs [3].

Transforming growth factor-β activated kinase 1 (TAK1, MAP3K7) is a protein kinase that regulates cell death and survival through activation of nuclear factor kappa-light-chain-enhancer of activated B cells (NF-κB) [4]. NF-κB is of critical importance in the pathogenesis of MM [5]. Targeting TAK1 has recently gained attention as a possible antimyeloma strategy [6–8]. We have previously shown that TAK1-inhibitors are cytotoxic to human myeloma cell lines patient cells [9]. To test whether TAK1-inhibitors also had an anti-MM effect in vivo we treated Vk*MYC MM mice with two different concentrations of the TAK1- inhibitor 5Z-7-oxozeaenol (5Z-7) and four different concentrations of the TAK1-inhibitor NG25. There were tendencies towards increased survival for both types of inhibitors, but only NG25 showed significant results. However, when measuring tumor load, no differences in burden of disease were observed. In conclusion, TAK1-inhibitors does not prevent disease in the Vκ*MYC model of MM.

## Main text

### Materials and methods

Experiments were performed after approval from the Norwegian Food Safety Authority (FOTS ID 21,756). The Norwegian Food Safety Authority works according to IACUC standards. All experiments were performed at the Department of comparative medicine, NTNU, Trondheim NO. The Vκ*MYC 12,653 model was kindly provided by Dr. Marta Chesi, Mayo Clinic AZ, USA. The initial generation and characterization of the Vκ*MYC mice have been described previously [10].

C57BL/6 J mice were chosen due to their compliancy with the Vκ*MYC model. Female mice were purchased from Janvier at 4 weeks of age and allowed to acclimatise for 4 weeks in the inhouse animal facility where experiments would be performed. Female animals were chosen to avoid fighting. At 8 weeks of age, the mice were intravenously injected with 2 million cells in 150 $\mu$L PBS from a single-cell suspension prepared from mice spleens containing 10% myeloma cells and previously cryopreserved in 10% DMSO/90% FCS. Hence, each mouse was injected with a total of 200 000 Vκ*MYC myeloma cells. All animals included in the experiment were injected with tumour. Treatment groups were the experimental units. Animals were randomly marked and allocated to groups prior to first tumour measurement. To avoid co-founders’ groups were kept in mixed cages – each cage containing one animal from each group, and treatment order was random. All personnel involved in injections were aware of group allocation, whereas animal facility personnel responsible for animal supervision were blinded. Exclusion criteria was if animals did not develop tumour (M-spike levels equal or higher than serum albumin levels).

To monitor tumour burden, M-spike levels were measured weekly starting week 4 post injection. Blood samples were collected by saphenous vein blood sampling. M-spike levels were measured by capillary electrophoresis with the Sebia Capillarys 2 instrument. Tumour onset was defined as when M-spike levels were equal to or higher than serum albumin levels.

Mice were then interperitoneally injected with Dimethyl Sulfoxide (DMSO, Sigma-Aldrich, D2650), 5Z-7-oxozeaenol (5Z-7, Sigma-Aldrich, O9890)), or NG25 (MedChemExpress, Monmouth Junction, NJ, USA, HY-15,434) on day 1–7 and day 12. In addition, spleen size was measured by weighing the spleen postmortem.

Survival was measured as days from start of treatment to death, or to sacrifice due to humane endpoint score. Spleen was isolated *postmortem* for weight analysis. For humane endpoint evaluation a standard score sheet was used according to guidelines from the NTNU animal facility (Department of comparative medicine) and the Norwegian Food Safety Authorities (Additional file 1).

Euthanasia was performed as follows: individual animals were inserted in a flow chamber containing to 5% isoflurane in 60% nitrous oxide and 35% oxygen for at least 5 min followed by cervical dislocation.

Statistical analysis was performed using the GraphPad Prism 9 software (GraphPad Software Inc., La Jolla, CA, USA). The tests used were Mantel-Cox log rank test, two-way ANOVA with Tukey’s multiple comparison test, or Kruskal-Wallis test with Dunn’s multiple comparison test. A P-value of < 0.05 was rendered significant for all comparisons.

### Results

#### NG25 prolong survival in a Vκ*MYC MM mouse model

**Fig. 1:**
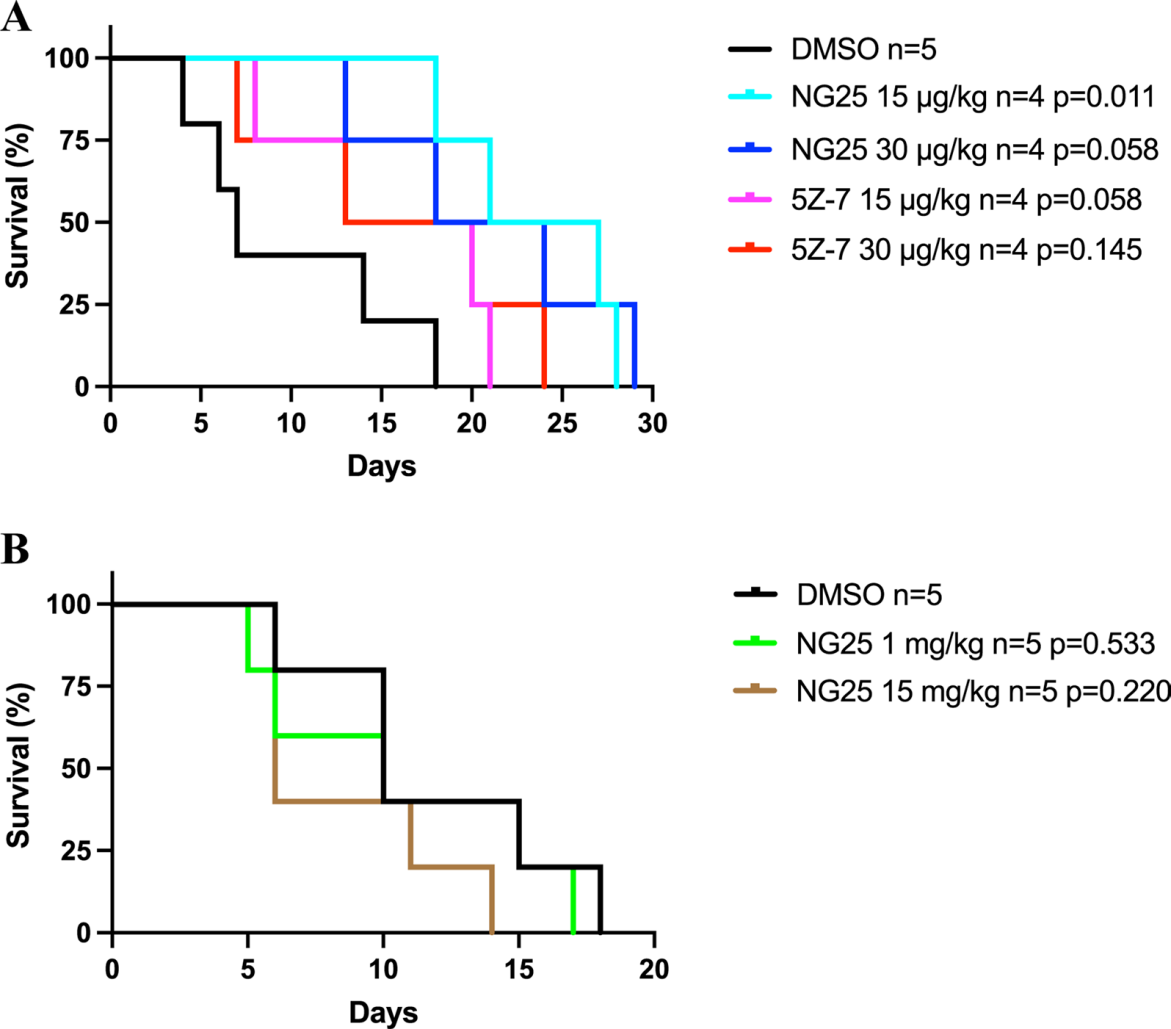
Survival after start of treatment in the Vk*MYC 12653 MM mouse model injected with TAK1-inhibitors. **A** Kaplan-Meier survival analysis for mice injected with vehicle (DMSO), 15 g/kg NG25, 30 g/kg NG25, 15 g/kg 5Z-7, or 30 g/kg 5Z-7. Five animals were designated to each group, and one animal was excluded for each group due to lack of tumor development. Final group size (n) is given for each group. **B**Kaplan-Meier survival analysis for mice injected with DMSO as control, 1 mg/kg NG25, and 15 mg/kg NG25. Five animals were designated to each group, and final group size (n) is given for each group. P-values are calculated with Mantel-Cox log rank test, and a P-value of <0.05 was rendered significant

 We tested two different types of TAK1-inhibitors, NG25 and 5Z-7, in the Vκ*MYC MM mouse model. NG25 in dosage of 15 $\mu$g/kg showed significant effect on survival compared with the control mice treated with DMSO. NG25 in dosage 30 $\mu$g/kg and 5Z-7 in dosages 15 $\mu$g/kg and 30 $\mu$g/kg showed a tendency towards increased survival, but these were not statistically significant (Fig. 1A). To further investigate a potential effect of NG25 we performed an additional experiment with 1 mg/kg and 15 mg/kg NG25. There was no difference in survival between NG25-treated and DMSO-treated animals in this experiment (Fig. 1B). In sum, NG25 prolonged survival in the Vκ*MYC MM mouse model but the general effect of TAK1-inhibition on survival was limited.

#### TAK1-inhibitor treatment had no effect on burden of disease in the Vκ*MYC MM mouse model

**Fig. 2:**
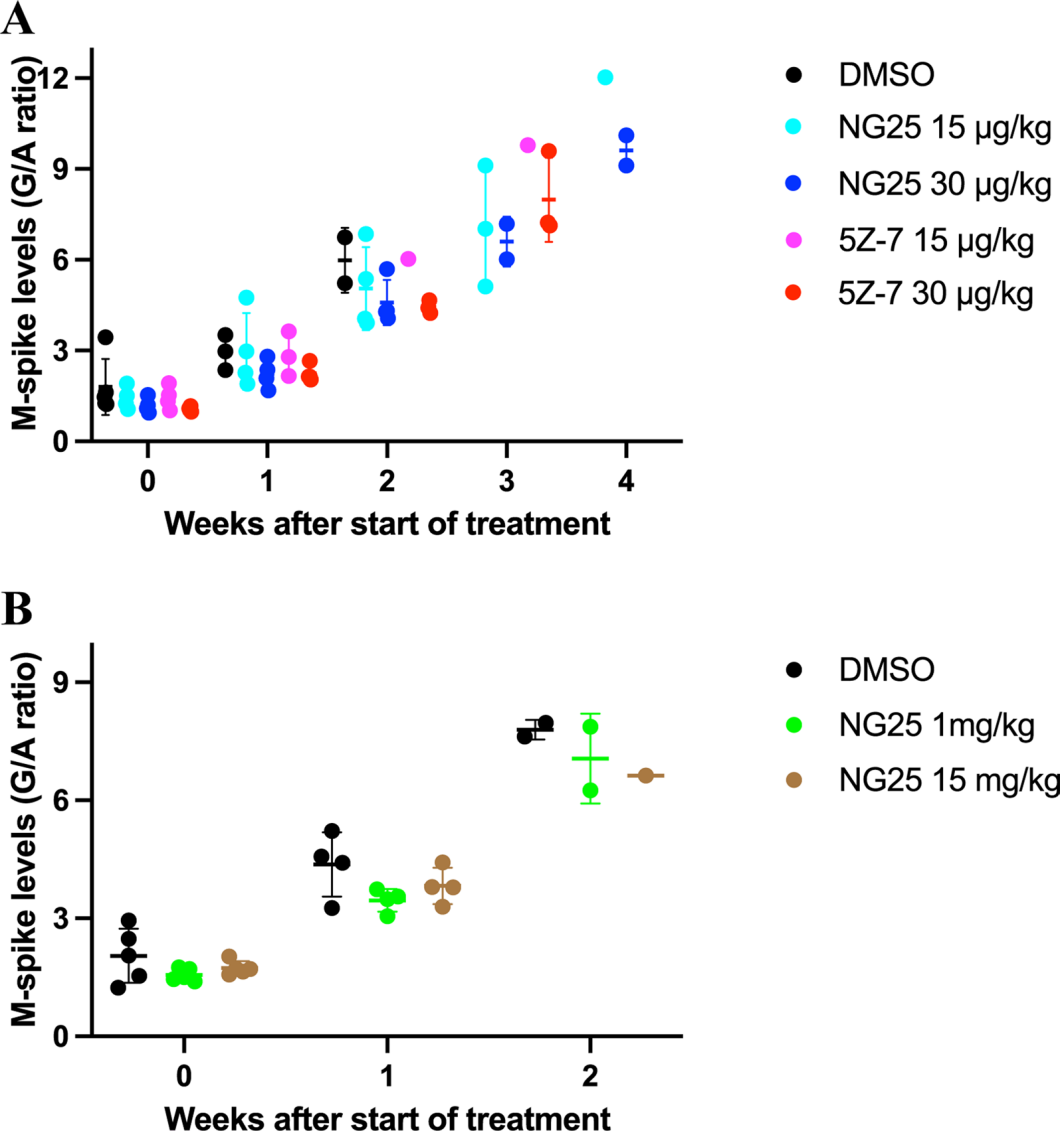
M-spike levels (gamma/album (G/A) ratio) in the Vκ*MYC 12,653 MM mouse model injected with TAK1-inhibitors. **A** Mice injected with vehicle (DMSO), 15 $\mu$g/kg NG25, 30 $\mu$g/kg NG25, 15 $\mu$g/kg 5Z-7, or 30 $\mu$g/kg 5Z-7. No significant differences were observed when treatment groups were compared with vehicle in the same week (Two-way ANOVA with Tukey´s multiple comparisons test). **B** Mice injected with vehicle (DMSO), 1 mg/kg NG25, or 15 mg/kg NG25. No significant differences were observed when treatment groups were compared with vehicle in the same week (Two-way ANOVA with Tukey´s multiple comparisons test). A P-value of < 0.05 was rendered significant. Dots are individual animals

**Fig. 3:**
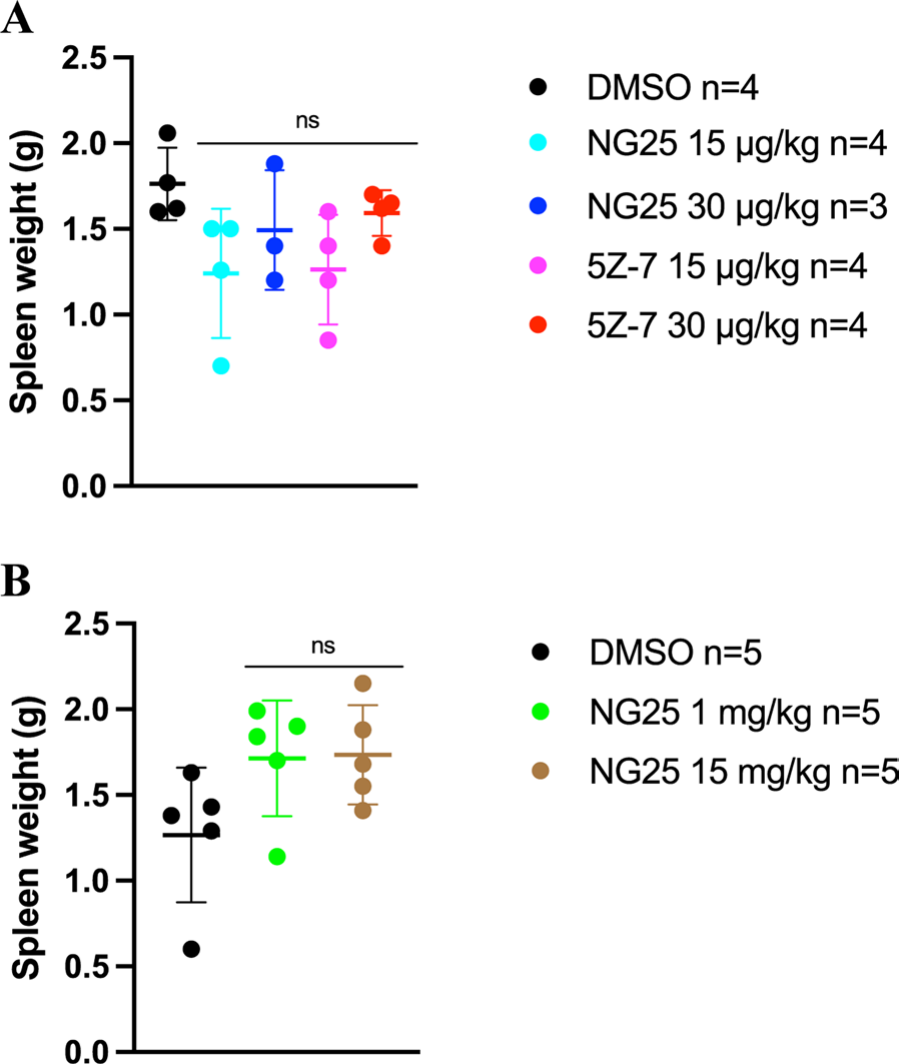
Spleen weight postmortem in the Vκ*MYC 12,653 MM mouse model. **A** Mice injected with vehicle (DMSO), 15 $\mu$g/kg NG25, 30 $\mu$g/kg NG25, 15 $\mu$g/kg 5Z-7, or 30 $\mu$g/kg 5Z-7. No significant differences were observed when treatment groups were compared with vehicle in the same week (Kruskal-Wallis test with Dunn´s multiple comparisons test). **B** Mice injected with vehicle (DMSO), 1 mg/kg NG25, or 15 mg/kg NG25. No significant differences were observed when treatment groups were compared with vehicle in the same week (Kruskal-Wallis test with Dunn´s multiple comparisons test). P-value < 0.05 was rendered significant

 To evaluate whether TAK1-inhibitors affected burden of disease we measured M-spike levels during treatment and measured spleen weight *postmortem*. We observed no differences in M-spike levels between treatment groups (Fig. 2). In addition, we observed splenomegaly in the Vκ*MYC animals, but spleen weight was not affected by TAK1-inhibitors (Fig. 3). Taken together, these findings suggest that TAK1-inhibitors have no effect on burden of disease in a Vκ*MYC MM mouse model.

### Discussion

In this study, we investigated the in vivo effect of TAK1 inhibitors NG25 and 5Z-7 in the Vκ*MYC 12,653 model of MM. 15 µg/kg NG25 prolonged survival, whereas this effect was not observed when dosages were increased. 5Z-7 did not give a significant prolongation on survival. Neither treatment had any effect on disease burden, as measured by M-spike levels.

Survival data showed a clear trend towards prolonged survival for when mice were treated with 15 or 30 µg/kg TAK1-inhibitors. Increasing group size might have rendered more treatments statistically significant. However, as treatments did not reduce tumour burden, the data were not sufficiently robust to repeat the experiments with larger group sizes.

Treatment with 5Z-7 at 20 mg/kg twice a week has been reported to reduce MM growth enhancement in immobilized hind legs [6]. 5Z-7 at 20 mg/kg every other day for 14 days has been shown in vivo to suppress MM tumour growth, serum IgG2b levels, and bone destruction. In the same study, the MM mouse model used was prepared by intra-tibial inoculation of mouse luciferase-transfected 5TGM1 MM cells to ICR nu/nu mice at 4–6 weeks old. The treatment was started 6 days after injection of tumour cells [8]. In our study treatment was started at least 4 weeks after injection of tumour cells, when tumour burden was more comparable to that at time of diagnosis for patients. Furthermore, our study differs from the other two mentioned studies as these do not report any values regarding survival of the mice. In conclusion, the in vivo effects of TAK1-inhibitors in MM are not consistent.

To determine whether TAK1-inhibitors are candidates as MM-treatment it must be clarified whether the effects of TAK1-inhibitors on human MM cells are species-specific, or an in vitro effect.

## Limitations

Limitations of this study include the limited number of animals used, a limited number of different dosages used, and the limited treatment regimen. In addition, the fact that only one mouse model was used is also a weakness of the study. To make clearer conclusions, further studies could include a larger number of animals and more than one mouse model.

## Supplementary Information

**Additional file 1**: Score sheet for assessment of humane end point.

## Data Availability

The datasets and material used and analysed in the current study are available from the corresponding author on reasonable request.

## Abbreviations

5Z-7: 5Z-7-oxozeaenol
MM: Multiple myeloma
TAK1: Transforming growth factor-β activated kinase 1
NF-κB: Nuclear factor kappa-light-chain-enhancer of activated B cells
